# The MMTV-Wnt1 murine model produces two phenotypically distinct subtypes of mammary tumors with unique therapeutic responses to an EGFR inhibitor

**DOI:** 10.1242/dmm.037192

**Published:** 2019-07-05

**Authors:** Adam D. Pfefferle, David B. Darr, Benjamin C. Calhoun, Kevin R. Mott, Jeffrey M. Rosen, Charles M. Perou

**Affiliations:** 1 Department of Pathology and Laboratory Medicine, University of North Carolina, Chapel Hill, NC 27599, USA; 2 Lineberger Comprehensive Cancer Center, University of North Carolina, Chapel Hill, NC 27599, USA; 3 Department of Genetics, University of North Carolina, Chapel Hill, NC 27599, USA; 4 Department of Molecular and Cellular Biology, Baylor College of Medicine, Houston, TX 77030, USA

**Keywords:** Breast cancer, MMTV-*Wnt1*, Genetically engineered mouse model, EGFR inhibitor

## Abstract

The Wnt gene family encodes an evolutionarily conserved group of proteins that regulate cell growth, differentiation and stem cell self-renewal. Aberrant Wnt signaling in human breast tumors has been proposed as a driver of tumorigenesis, especially in the basal-like tumor subtype where canonical Wnt signaling is both enriched and predictive of poor clinical outcomes. The development of effective Wnt-based therapeutics, however, has been slowed in part by a limited understanding of the context-dependent nature with which these aberrations influence breast tumorigenesis. We previously reported that MMTV-Wnt1 mice, an established model for studying Wnt signaling in breast tumors, develop two subtypes of tumors by gene expression classification: Wnt1-Early^Ex^ and Wnt1-Late^Ex^. Here, we extend this initial observation and show that Wnt1-Early^Ex^ tumors exhibit high expression of canonical Wnt, non-canonical Wnt, and EGFR signaling pathway signatures. Therapeutically, Wnt1-Early^Ex^ tumors showed a dynamic reduction in tumor volume when treated with an EGFR inhibitor. Wnt1-Early^Ex^ tumors had primarily Cd49f^pos^/Epcam^neg^ FACS profiles, but it was not possible to serially transplant these tumors into wild-type FVB female mice. Conversely, Wnt1-Late^Ex^ tumors had a bloody gross pathology, which was highlighted by the presence of ‘blood lakes’ identified by H&E staining. These tumors had primarily Cd49f^pos^/Epcam^pos^ FACS profiles, but also contained a secondary Cd49f^pos^/Epcam^neg^ subpopulation. Wnt1-Late^Ex^ tumors were enriched for activating *Hras1* mutations and were capable of reproducing tumors when serially transplanted into wild-type FVB female mice. This study definitively shows that the MMTV-Wnt1 mouse model produces two phenotypically distinct subtypes of mammary tumors that differ in multiple biological aspects including sensitivity to an EGFR inhibitor.

## Abstract (precis)

**Summary:** The MMTV-Wnt1 mouse model produces two phenotypically distinct subtypes of mammary tumors. Importantly, these subtypes differ in their therapeutic response to erlotinib, an EGFR inhibitor.

## INTRODUCTION

The mammalian breast is a unique organ capable of dynamic morphological and physiological change during organogenesis, puberty, pregnancy, lactation and involution (Gjorevski and Nelson, 2011). These processes are supported by a breast morphology that can be subdivided into four primary compartments: the stroma, the basement membrane, the basal layer and the luminal layer (Roarty and Rosen, 2010; Visvader, 2009). Within each of these compartments reside specific cell types that together form a mammary cell hierarchy (Van Keymeulen et al., 2011; Santagata et al., 2014; Visvader and Stingl, 2014). Specifically, the stroma consists primarily of fibroblasts, adipocytes and immune cells (Arendt et al., 2010; Visvader, 2009). The basal layer is enriched for myoepithelial cells and mammary stem cells (MaSC) (Woodward et al., 2005) and the luminal layer contains a combination of estrogen receptor (ER)-positive and ER-negative mature luminal cells (Visvader, 2009).

Each cell within this hierarchy has developed specialized functions to support the necessary changes that will occur over a woman's lifetime. These processes include important elements of paracrine signaling to transmit signals across the different mammary compartments to specific recipients (Rosen and Roarty, 2014). The Wnt family is an evolutionarily conserved group of proteins that promote autocrine and paracrine signal transduction through at least five different pathways (Roarty and Rosen, 2010). The canonical Wnt pathway signals through Frizzled (Fzd) and low-density lipoprotein (LDL)-receptor-related (Lrp) co-receptors (Lrp5 and Lrp6) to activate β-catenin transcriptional regulation of key genes (Roarty and Rosen, 2010), such as *c-Myc* (He et al., 1998), *c-Jun* (Mann et al., 1999) and *Vegf* (Zhang et al., 2001). The other Wnt-regulated pathways are collectively referred to as non-canonical Wnt signaling. These include calcium and planar cell polarity signaling through Fzd receptors, Jnk signaling through the Ror2 receptor and Src signaling through the Ryk receptor (Roarty and Rosen, 2010). Although these pathways are commonly described from a cell autonomous perspective, complex signaling patterns emerge when paracrine signaling is considered (Rosen and Roarty, 2014). In addition, there are 19 Wnt ligands and 10 Fzd receptors. When taking into account co-receptors and cell type-specific expression patterns (Lim et al., 2010; Kendrick et al., 2008), a large number of combinations are possible. Given the importance of Wnt signaling for controlling cell growth, differentiation and stem cell self-renewal (Anastas and Moon, 2013), a research emphasis has been placed on better understanding these Wnt signaling pathways.

One area of particular focus has been determining how aberrant Wnt signaling influences breast tumor formation and progression. Breast cancer is a heterogeneous disease that can be segregated into at least six distinct intrinsic subtypes based on gene expression profiles: basal-like, claudin-low, HER2-enriched, luminal A, luminal B and normal-like (Perou et al., 2000; Prat et al., 2010; Cancer Genome Atlas Network, 2012). Interestingly, canonical Wnt signaling is enriched in basal-like breast tumors (Khramtsov et al., 2010). These patients also tend to have a poor clinical outcome (Khramtsov et al., 2010), suggesting Wnt signaling as a potential therapeutic target (Anastas and Moon, 2013). Unlike colorectal cancer where inappropriate Wnt pathway activation is associated with gene mutations (Segditsas and Tomlinson, 2006), mutations affecting Wnt-associated genes are uncommon in breast tumors (Cancer Genome Atlas Network, 2012). Instead, activation in breast tumors is proposed to occur through the downregulation of negative Wnt pathway regulators, such as secreted frizzle-related proteins (Suzuki et al., 2008; Veeck et al., 2008). Although these pathways have been examined extensively, more research is needed to fully untangle the complex behavior of these signaling molecules. For instance, the molecular mechanisms that explain how paracrine Wnt signaling can induce growth of some tumors and inhibit growth in others have remained elusive (Green et al., 2013).

Genetically engineered mouse models are a useful resource for studying mammary tumors *in vivo* under genetically controlled and immune-competent conditions (Sharpless and Depinho, 2006). MMTV-Wnt1 mice are an established model for investigating aberrant Wnt signaling in breast tumors (Li et al., 2000; Nusse and Varmus, 1982). These tumors are composed of mixed-lineage subclonal populations, having features of both luminal and basal epithelial cells (Cleary et al., 2014). In a subset of MMTV-Wnt1 tumors, cooperation between both subclonal populations might be required for efficient tumor propagation (Cleary et al., 2014), highlighting this model as a tool for studying Wnt paracrine signaling and intratumoral heterogeneity (Zhang et al., 2015). On the basis of gene expression profiling, we previously reported that MMTV-Wnt1 mice develop two subtypes/classes of tumors (Pfefferle et al., 2013), a finding that is surprisingly underappreciated in the vast literature on this model. Here, we investigate the significance of our earlier observation and show that that these two classes of tumors have distinct phenotypes.

## RESULTS

**Fig. 1.**
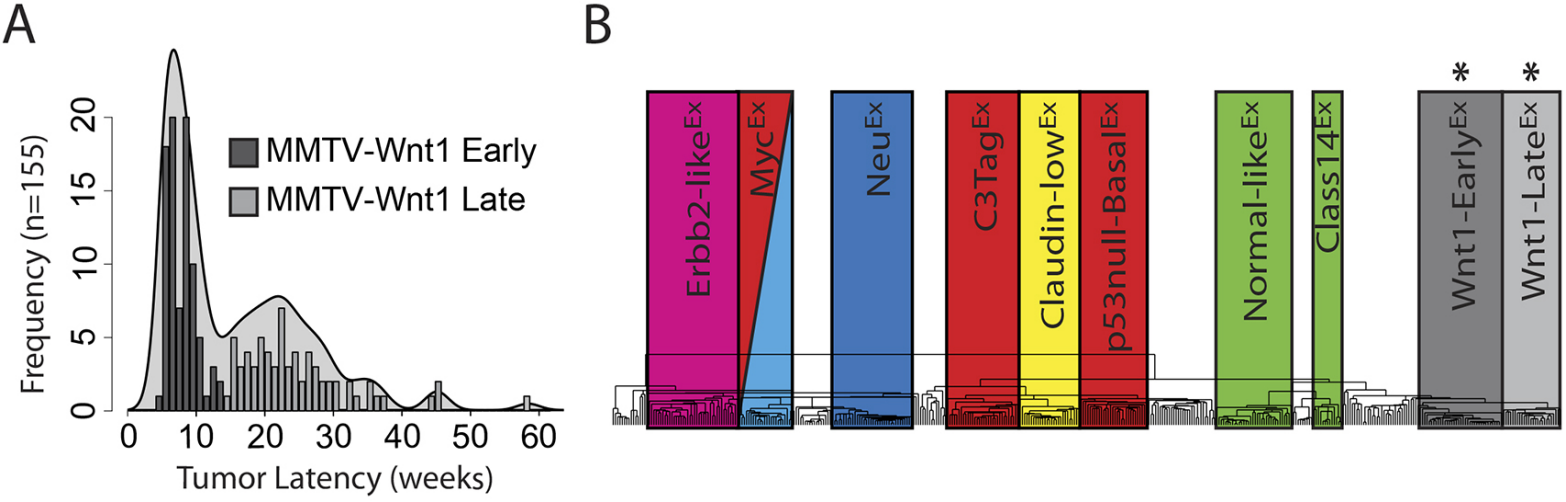
**The MMTV-Wnt1 model produces two classes of mammary tumors with distinct primary tumor latencies.** (A) MMTV-Wnt1 primary tumor latency histogram by week with a superimposed density plot. The cut-off between the early and late tumor subtypes was identified to be around 14-15 weeks based on numerous lines of evidence, which is highlighted here by the dip in the density plot. (B) Dendrogram of a hierarchical cluster of all murine tumors in our data set using a previously defined intrinsic gene list (Pfefferle et al., 2013). Boxes correspond to previously defined murine intrinsic subtypes/classes, with the asterisks highlighting the two classes that are enriched for MMTV-Wnt1 tumors (Pfefferle et al., 2013).

Although the Wnt family has been studied extensively from both a developmental and oncological perspective, the complexity of this pathway has hindered a complete understanding of the molecular mechanisms that regulate cell growth, differentiation and stem cell self-renewal (Anastas and Moon, 2013). The MMTV-Wnt1 murine model is attractive for studying aberrant Wnt signaling in breast carcinoma (Li et al., 2000; Nusse and Varmus, 1982). Interestingly, we find that these mice have a broad distribution of tumor latencies, developing tumors as early as 5 weeks and as late as 58 weeks of age (Fig. 1A). A histogram of 155 tumor latencies produces a bimodal distribution, with an ‘early’ local maximum around 6.5 weeks and a ‘late’ local maximum around 22.5 weeks based upon a density plot. Importantly, tumor latency did not correlate with mouse birth order, suggesting these latency differences are not the result of genetic drift or some other non-biological variable (Fig. S1). Although no differences were observed on a DNA copy number level between early versus late tumors (Fig. S2), gene expression profiling found that these Wnt-Early^Ex^ and Wnt1-Late^Ex^ tumors have distinct expression and pathological features (Pfefferle et al., 2013) (Fig. 1B). This is unlikely to be an artifact of tumor location, as both Wnt1-Early^Ex^ and Wnt1-Late^Ex^ tumors sporadically formed in all murine mammary glands. Given these findings, we performed an in-depth comparison of these two Wnt1 murine tumor classes to further our understanding of the biological significance of Wnt signaling in breast cancer.

### Wnt1-Early^Ex^ and Wnt1-Late^Ex^ tumors have distinct gross pathology and histopathology

**Fig. 2.**
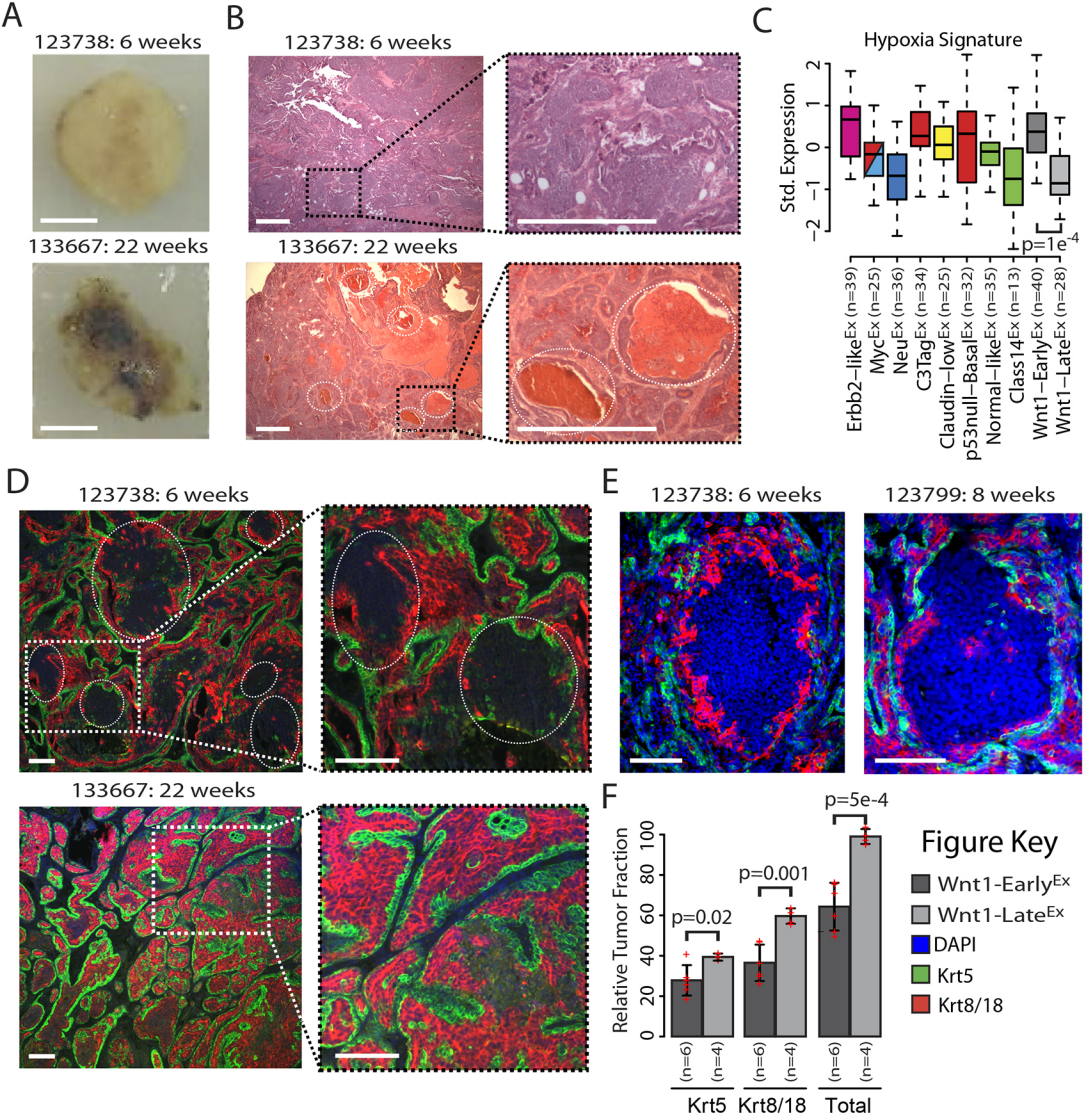
**The MMTV-Wnt1 model produce two classes of mammary tumors with distinct gross pathology and histology features.** (A) Gross pathology of representative Wnt1-Early^Ex^ and Wnt1-Late^Ex^ tumors. Scale bar: 0.5 mm. (B) H&E staining of representative Wnt1-Early^Ex^ and Wnt1-Late^Ex^ tumors. The dotted circles highlight blood lake regions. Scale bar: 5 mm. (C) Standardized expression of a hypoxia gene signature (Hu et al., 2009) across mouse classes, as displayed by box and whisker plots. The box represents the 25%, 50% and 75% percentiles. The whiskers represent either 1.5× interquartile range (IQR) or the most extreme data point if less than 1.5× IQR. The *P*-value was calculated using an unpaired *t*-test. (D) DAPI (blue), Krt5 (green; a marker of basal cells) and Krt8/18 (red; a marker of luminal cells) staining of representative Wnt1-Early^Ex^ and Wnt1-Late^Ex^ tumors. Scale bar: 0.1 mm. (E) DAPI, Krt5 and Krt8/18 staining of representative Wnt1-Early^Ex^ tumors. Scale bar: 0.1 mm. (F) Relative tumor fraction of Krt5-positive and Krt8/18-positive cells. Each measurement originated from an independent primary tumor and is displayed as a cross. The error bars represent 1 s.d. *P*-values were calculated using unpaired *t*-tests.

In addition to being classified into different molecular expression subtypes/classes, Wnt1-Early^Ex^ and Wnt1-Late^Ex^ tumors were also found to have distinct gross pathological features. Specifically, 85.7% of Wnt1-Early^Ex^ tumors tended to have a dense cellular morphology and be more resistant to incision, whereas 80% of Wnt1-Late^Ex^ tumors appeared to be more vascular and often filled with pockets of blood (Fig. 2A and Fig. S3). Importantly, these characteristics were found to be irrespective of tumor size at the time of collection, indicating that these observations were not a technical artifact but inherent to the tumors themselves. Hematoxylin and eosin (H&E)-stained slides from nine MMTV-Wnt1 tumors were reviewed by a breast pathologist in a non-blinded fashion. The primary architectural pattern and secondary patterns or other prominent findings (e.g. blood lakes, necrosis) were recorded (Table S1) (Cardiff et al., 2000; Munn et al., 1995). Although there was some overlap in the histological appearance of Wnt1-Early^Ex^ and Wnt1-Late^Ex^ tumors, papillary architecture and blood lakes (dashed circles in Fig. 2B and Fig. S4) appeared to be more common in the Wnt1-Late^Ex^ tumors. The Wnt1-Early^Ex^ tumors showed more solid and cord-like (or trabecular) patterns, as well as areas with glandular architecture and central necrosis. These different vascular traits led us to hypothesize that Wnt1-Early^Ex^ tumors might be more hypoxic than Wnt1-Late^Ex^ tumors. In support of this, a vascular endothelial growth factor (VEGF)/hypoxia gene signature (Hu et al., 2009) is highly expressed in Wnt1-Early^Ex^ tumors, but is expressed at lower levels in Wnt1-Late^Ex^ tumors (Fig. 2C).

It is well documented that MMTV-Wnt1 tumors are composed of mixed-lineage subclonal populations, having features of both luminal and basal cells (Cleary et al., 2014). To investigate the relative fraction of these subclonal populations, immunofluorescence staining was performed using antibodies against Krt5 (a marker of basal cells) and Krt8/18 (a marker of luminal cells). Consistent with the literature, both Wnt1-Early^Ex^ and Wnt1-Late^Ex^ tumors stained positive for both cell populations (Fig. 2D and Fig. S5). Unlike Wnt1-Late^Ex^, Wnt1-Early^Ex^ tumors had distinct regions that did not stain positive for either Krt5 or Krt8/18 (Fig. 2D,E,F and Fig. S5). These areas did stain positive for DAPI, indicating that there are cells within these regions. When measuring the relative tumor fractions of these populations, it was observed that Wnt1-Late^Ex^ tumors contained a higher fraction of Krt5-positive cells (*P*=0.02) and Krt8/18-positive cells (*P*=0.001) than Wnt1-Early^Ex^ tumors (Fig. 2F). Combined, these two cell fractions comprised about 100% of Wnt1-Late^Ex^ tumors. For Wnt1-Early^Ex^ tumors, these two fractions accounted for only about 65% of the tumor, with the remaining 35% consisting of regions/cells that did not stain positive for either Krt5 or Krt8/18; the identify of these non-staining cell type(s) is unknown.

### Wnt1-Early^Ex^ tumors are enriched for canonical and non-canonical Wnt pathway signatures

**Fig. 3.**
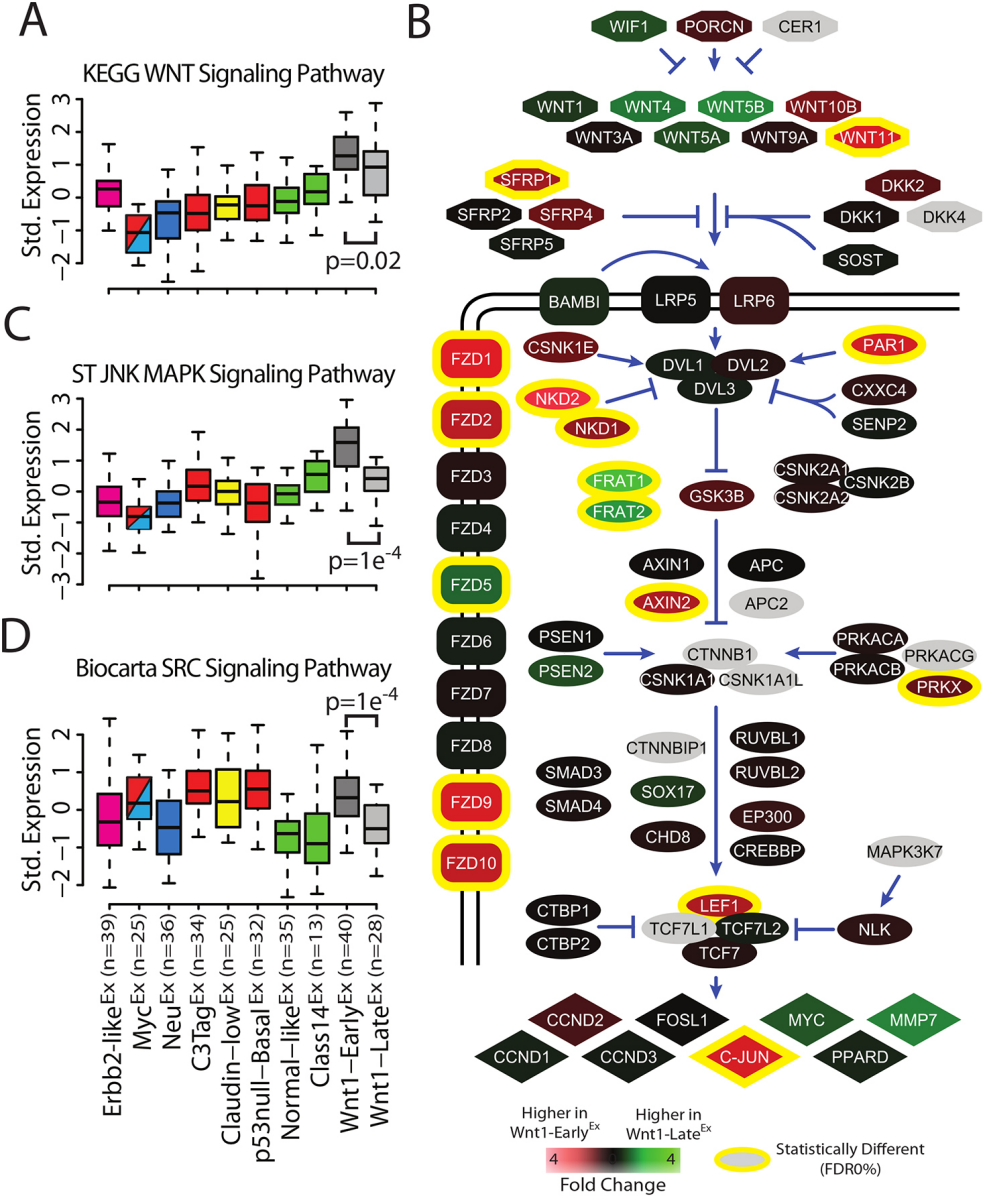
**Wnt-Early^Ex^ tumors show expression of Wnt-associated pathway signatures.** (A) Standardized expression of the KEGG Wnt signaling pathway signature across mouse class, as displayed by box and whisker plots. The *P*-value was calculated using an unpaired *t*-test. (B) Schematic of the KEGG Wnt signaling pathway. False discovery rate (FDR) was calculated using a two-class SAM analysis on the microarray data set. (C) Standardized expression of the ST JNK MAPK signaling pathway signature across mouse class, as displayed by box and whisker plots. The *P*-value was calculated using an unpaired *t*-test. (D) Standardized expression of the Biocarta SRC signaling pathway signature across mouse class, as displayed by box and whisker plots. The *P*-value was calculated using an unpaired *t*-test.

Wnt signal transduction can occur through several different molecular pathways, including canonical Wnt, Jnk and Src (Roarty and Rosen, 2010). To investigate these pathways in our mouse tumors, expression-based pathway gene signatures were used to estimate pathway activity (Pfefferle et al., 2013; Fan et al., 2011). As expected, several pathway signatures were significantly upregulated and downregulated in both Wnt1-Early^Ex^ and Wnt1-Late^Ex^ tumors (Tables S2, S3, and S4). As a positive control, the canonical KEGG Wnt signaling pathway was the most highly expressed in both Wnt1-Early^Ex^ and Wnt1-Late^Ex^ classes when compared with the other eight mouse classes (Fig. 3A); interestingly, this pathway signature is more highly expressed in Wnt1-Early^Ex^ than in Wnt1-Late^Ex^ tumors (*P*=0.02). This observation does not appear to be a result of variation in Wnt1 transgene expression, as there was no statistical difference between the Wnt1-Early^Ex^ and Wnt1-Late^Ex^ classes for the single Wnt1 probe (exon 4) in our combined murine data set (Fig. 3B). A closer investigation into the individual genes within the canonical KEGG Wnt signaling pathway identified several differentially expressed genes between Wnt1-Early^Ex^ and Wnt1-Late^Ex^ tumors (Fig. 3B). Canonical Wnt signaling occurs through Fzd and Lrp co-receptors (Roarty and Rosen, 2010). Fzd receptors *Fzd1*, *Fzd2*, *Fzd9* and *Fzd10* are more highly expressed in Wnt1-Early^Ex^ tumors, whereas *Fzd5* is more highly expressed in Wnt1-Late^Ex^ tumors (FDR 0%). In addition, the transcription factor *Lef1* and its target *c-Jun* (Mann et al., 1999), are more highly expressed in Wnt1-Early^Ex^ tumors (FDR 0%). These results are consistent with higher canonical Wnt pathway activity in Wnt1-Early^Ex^ tumors.

In addition to higher expression of the canonical KEGG Wnt signaling pathway signature, Wnt1-Early^Ex^ tumors have higher expression of non-canonical Wnt signaling pathway signatures: ST JNK MAPK signaling pathway (*P*=1.0e^−4^) (Fig. 3C) and Biocarta SRC signaling pathway (*P*=1.0e^−4^) (Fig. 3D). These results are intriguing because they suggest that Wnt1-Early^Ex^ tumors signal through both canonical and non-canonical Wnt pathways to a greater extent than Wnt1-Late^Ex^ tumors.

### Wnt1-Early^Ex^ tumors respond to an epidermal growth factor receptor inhibitor

**Fig. 4.**
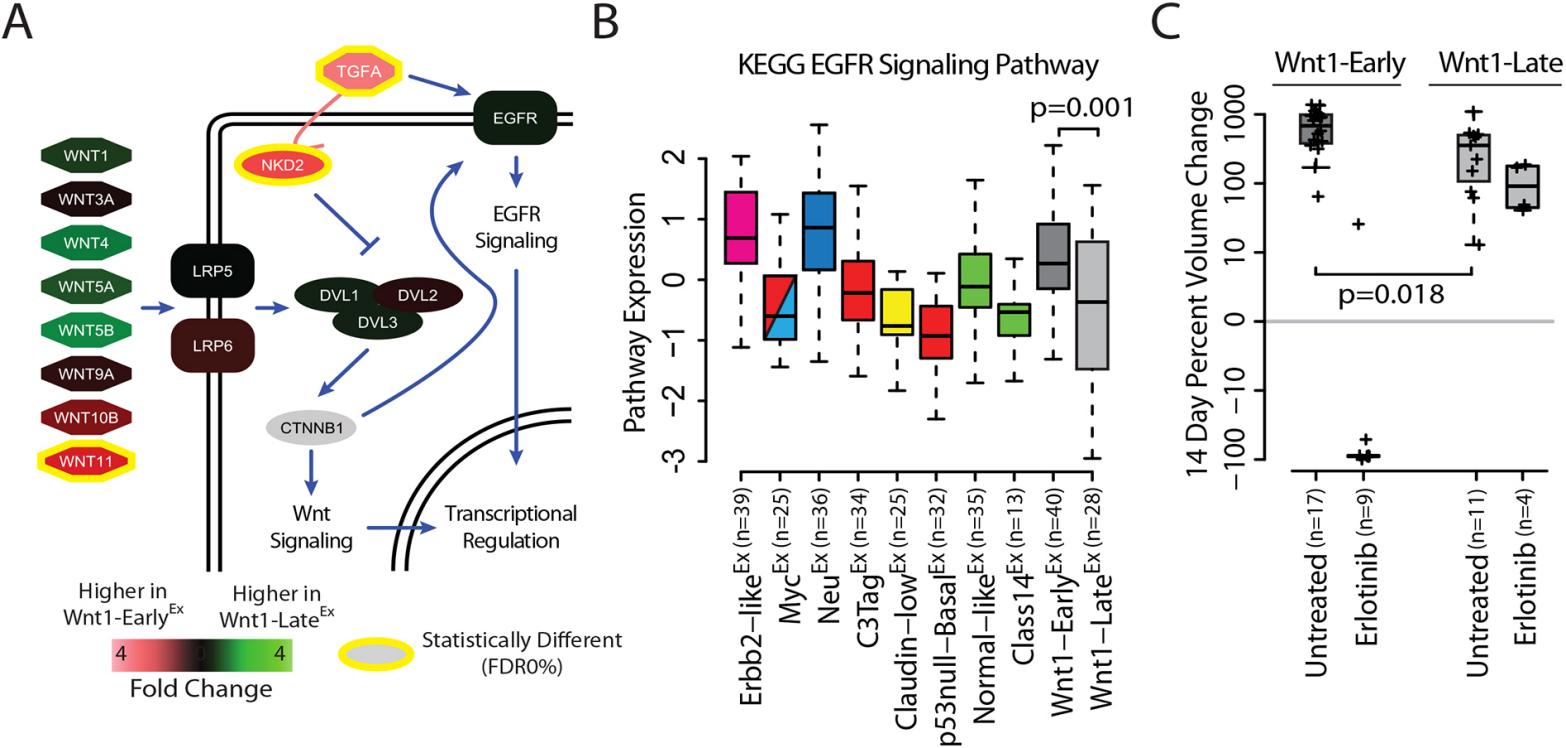
**Wnt-Early^Ex^ tumors respond to EGFR inhibitors.** (A) Schematic of WNT and EGFR pathway crosstalk. False discovery rate (FDR) was calculated using a two-class SAM analysis on the microarray data set. (B) Standardized expression of the KEGG EGFR signaling pathway signature across mouse class as displayed by box and whisker plots. The *P*-value was calculated using an unpaired *t*-test. (C) 14-day tumor response to erlotinib treatment, as displayed by box and whisker plots. Each measurement originated from an independent primary tumor and is displayed as a cross. The *P*-value was calculated using an unpaired *t*-test.

In addition to canonical and non-canonical signaling, Wnt-associated genes can also crosstalk with a variety of other signal transduction pathways (Collu et al., 2014; Nishita et al., 2000; Shackleford et al., 1993), including epidermal growth factor receptor (EGFR) signaling (Hu and Li, 2010). Specifically, naked cuticle 2 (NKD2) is capable of binding and shuttling transforming growth factor α (TGF-α) to the plasma membrane, which serves as an activating ligand of EGFR (Fig. 4A). Interestingly, both *Nkd2* and *Tgfα* are more highly expressed in Wnt1-Early^Ex^ tumors (FDR 0%). Given this observation, we hypothesized that Wnt1-Early^Ex^ tumors might have a greater degree of EGFR signaling than Wnt1-Late^Ex^ tumors. Consistent with this, the KEGG EGFR signaling pathway signature is more highly expressed in Wnt1-Early^Ex^ as compared with Wnt1-Late^Ex^ tumors (*P*=0.001) (Fig. 4B).

To determine the clinical importance of these findings, Wnt1-Early^Ex^ and Wnt1-Late^Ex^ tumors were randomized into one of two treatment groups: untreated or treated with erlotinib (an EGFR inhibitor) (Table S5). A comparison of the untreated groups found that Wnt1-Early^Ex^ tumors proliferate faster than Wnt1-Late^Ex^ tumors (*P*=0.018). As hypothesized, Wnt1-Early^Ex^ tumors had a median tumor regression of 90% when treated with erlotinib at the end of the 2 week treatment period (Fig. 4C). Wnt1-Late^Ex^ tumors, however, continued to progress with erlotinib treatment with a median tumor growth of 109%. These results indicate that Wnt1-Early^Ex^ tumors were therapeutically more responsive to erlotinib than Wnt1-Late^Ex^ tumors.

### Wnt1-Early^Ex^ and Wnt1-Late^Ex^ tumors have distinct mammary subpopulation FACS profiles

Normal mammary gland physiology is supported by an underlying, complex cell hierarchy (Van Keymeulen et al., 2011; Santagata et al., 2014; Visvader and Stingl, 2014). A simplistic model places the multipotent MaSC at the base of this hierarchy, having extensive, self-regenerative potential (Visvader, 2009). During mammary development, the MaSC has been proposed to divide asymmetrically to produce basal/myoepithelial cells as well as luminal progenitors (LumProg), which have more restricted proliferative and differentiation capabilities (Visvader, 2009). LumProg cells are capable of further differentiation into mature luminal (MatureLum) cells, such as ER-positive ductal epithelium; cells of this epithelium have an even more limited proliferative potential and some are terminally differentiated (Visvader, 2009).

**Fig. 5.**
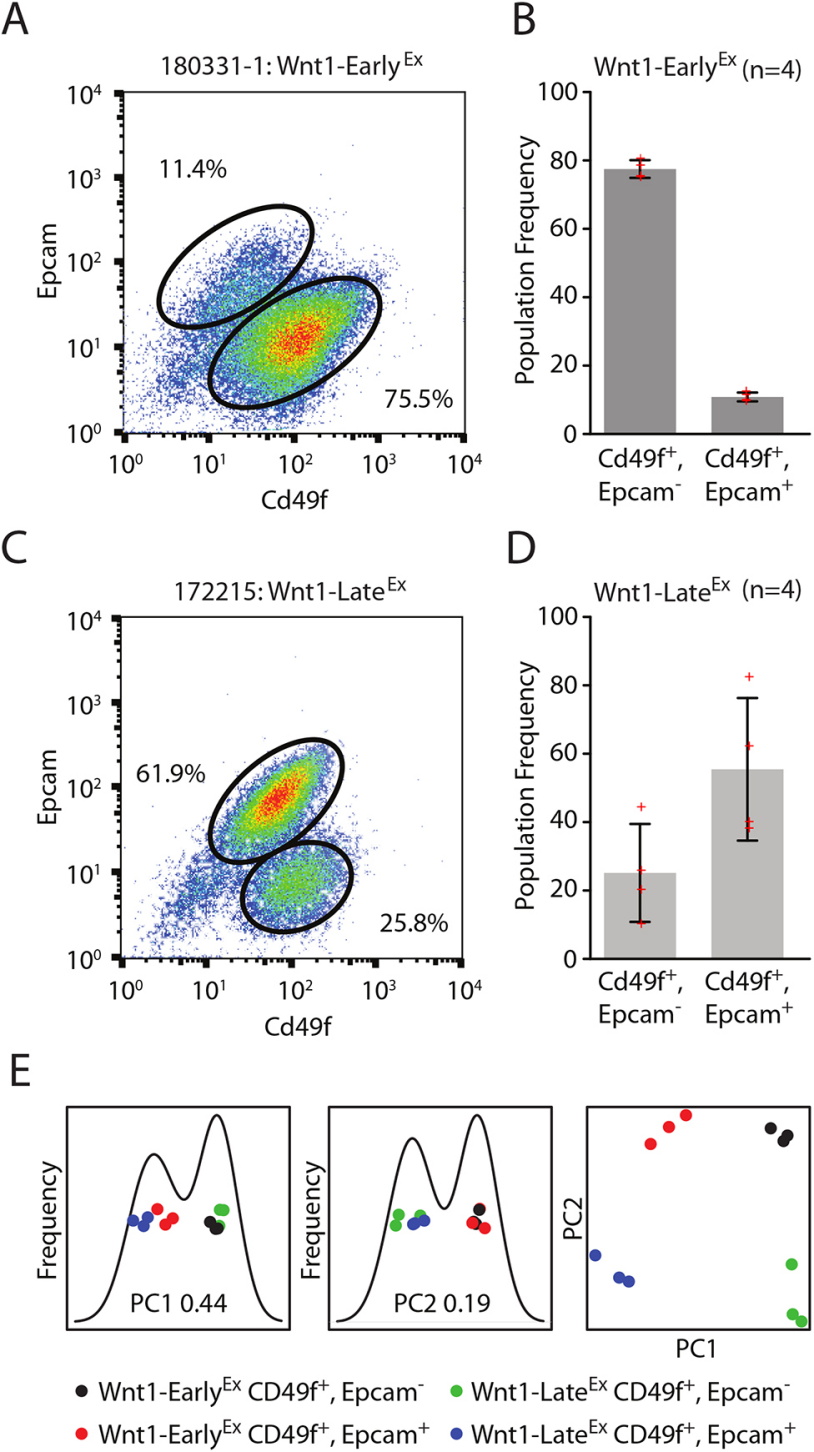
**Wnt-Early^Ex^ and Wnt1-Late^Ex^ tumors share features with different normal mammary cell types.** (A) Cd49f/Epcam FACS profile of a representative Wnt1-Early^Ex^ tumor. (B) FACS population frequencies of Wnt1-Early^Ex^ tumors as measured from four independent primary tumors. Each measurement is represented as a cross. The error bars represent 1 s.d. (C) Cd49f/Epcam FACS profile of a representative Wnt1-Late^Ex^ tumor. (D) FACS population frequencies of Wnt1-Late^Ex^ tumors as measured from four independent primary tumors. Each measurement is represented as a cross. The error bars represent 1 s.d. (E) First two principle components of FACS-sorted Wnt1 tumor gene expression profiles.

MMTV-Wnt1 tumors might originate from several of the cell types within this mammary hierarchy. To determine if Wnt1-Early^Ex^ and Wnt1-Late^Ex^ tumors share features with any of these cell populations, four primary tumors from each class were analyzed by fluorescence-activated cell sorting (FACS) using antibodies against Cd49f and Epcam (Cleary et al., 2014; Pfefferle et al., 2015). FACS profiles of Wnt1-Early^Ex^ tumors consisted of two populations (Fig. 5A): the major (∼75%) epithelial cell population was Cd49f^pos^/Epcam^neg^, whereas the minor (∼10%) population was Cd49f^pos^/Epcam^pos^ (Fig. 5B). Normal human MaSCs are defined as having CD49f^pos^/Epcam^neg^ FACS profiles (Visvader, 2009), indicating that the majority of Wnt1-Early^Ex^ tumor cells share similar features with normal MaSCs. Although Wnt1-Late^Ex^ tumor FACS profiles also had two FACS populations, the frequencies were distinct from Wnt1-Early^Ex^ tumors (Fig. 5C). Specifically, the major (∼60%) epithelial cell population was Cd49f^pos^/Epcam^pos^, whereas the minor population (∼25%) was Cd49f^pos^/Epcam^neg^ (Fig. 5D). Normal human LumProg cells are defined as having CD49f^pos^/Epcam^pos^ FACS profiles (Visvader, 2009), indicating that the majority of Wnt1-Late^Ex^ tumor cells might share similar features with normal LumProg cells.

It is possible that the MMTV-Wnt1 model produces semi-homogeneous tumors (Pfefferle et al., 2013) simply because of intra-tumor variation in the frequencies of these two FACS populations and not because of differences between corresponding FACS populations across the two classes themselves. For example, this hypothesis would propose that the Wnt1-Early^Ex^ Cd49f^pos^/Epcam^neg^ population should be phenotypically the same as the Wnt1-Late^Ex^ Cd49f^pos^/Epcam^neg^ population. To address this issue, FACS was used to sort three tumors from each Wnt1 class into their corresponding populations, which were then analyzed using microarrays. A global transcriptomic comparison of these FACS populations using a principal component (PC) analysis highlights that the first PC separates the Cd49f^pos^/Epcam^neg^ population from the Cd49f^pos^/Epcam^pos^ population, irrespective of the Wnt1 tumor class from which they were derived (Fig. 5E). This observation is consistent with the proposed hypothesis that these FACS populations are phenotypically similar across classes, but the first PC only explains 44% of the variation. The second PC, which explains 19% of the variation, separates Wnt1-Early^Ex^ from Wnt1-Late^Ex^ tumors. Taken together, these results indicate that, although the corresponding FACS populations are highly similar across Wnt1-Early^Ex^ and Wnt1-Late^Ex^ tumors, they also have class/subtype-specific features.

### Both Wnt1-Late^Ex^ tumor FACS subpopulations have tumor-initiating potential

**Fig. 6.**
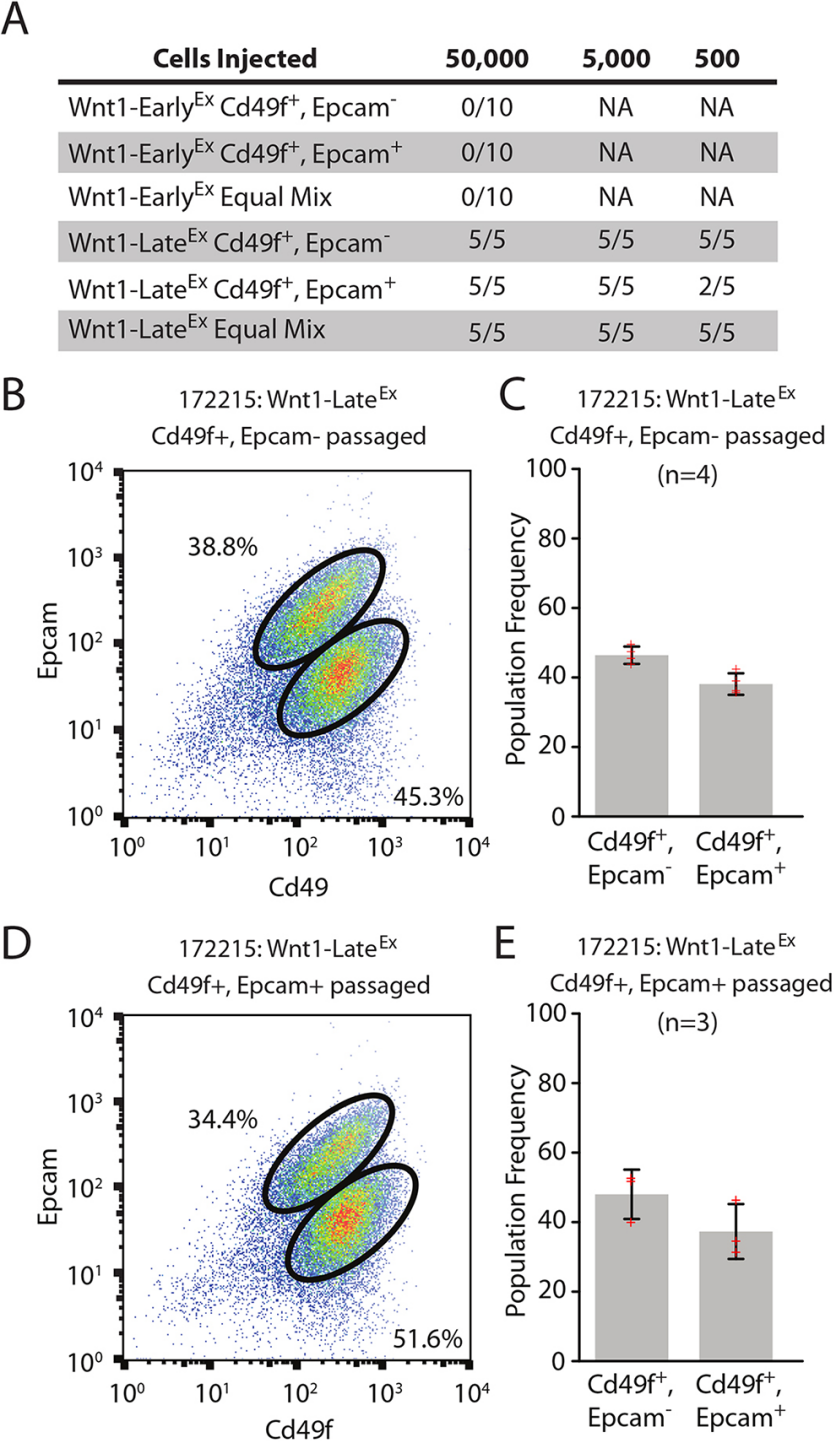
**Both Wnt1-Late^Ex^ tumor subpopulations have tumor-initiating potential.** (A) Limiting dilution cell transplantation assay. Three primary tumors (two Wnt1-Early^Ex^ and one Wnt1-Late^Ex^) were transplanted into five 6- to 8-week-old wild-type FVB female mice at each cell concentration and monitored for tumor growth over 120 days. (B) Cd49f/Epcam FACS profile of a representative Wnt1-Late^Ex^ CD49f^pos^/Epcam^neg^ passaged tumor. (C) FACS population frequencies of Wnt1-Late^Ex^ CD49f^pos^/Epcam^neg^ passaged tumors as measured from four independent transplanted tumors. Each measurement is represented as a cross. The error bars represent 1 s.d. (D) Cd49f/Epcam FACS profile of a representative Wnt1-Late^Ex^ CD49f^pos^/Epcam^pos^ passaged tumor. (E) FACS population frequencies of Wnt1-Late^Ex^ CD49f^pos^/Epcam^pos^ passaged tumors as measured from three independent transplanted tumors. Each measurement is represented as a cross. The error bars represent 1 s.d.

Given that Wnt1-Early^Ex^ tumors share features with normal MaSCs and that Wnt1-Late^Ex^ tumors share features with normal LumProg cells, we hypothesized that these two Wnt1 classes might have different tumor-initiating potential. To test this idea, three primary Wnt1 tumors (two Wnt1-Early^Ex^ and one Wnt1-Late^Ex^) were sorted using FACS into their subpopulations and a limiting dilution assay was performed in which each subpopulation was injected into the mammary pad of female FVB wild-type mice. In addition, a subset of MMTV-Wnt1 tumors requires both FACS populations for tumor growth (Cleary et al., 2014); therefore, a third cohort consisting of an equal mixture of each FACS subpopulation was also performed to investigate this possibility in our two classes. Interestingly, no fraction or combination of FACS fractions of the two Wnt1-Early^Ex^ tumors investigated were able to be serially transplanted into wild-type mice, giving rise to no tumors after injection with 50,000 cells (Fig. 6A). Although we are unable to definitively state that all Wnt1-Early^Ex^ tumors cannot be passaged based on two primary tumors, the data presented indicate that a subset of Wnt1-Early^Ex^ tumors are not able to be serially transplanted in female FVB wild-type mice. Conversely, all combinations of the one Wnt1-Late^Ex^ tumor investigated gave rise to tumors.

Tumors that arose from the individual Wnt1-Late^Ex^ FACS populations were then re-analyzed with FACS to investigate their tumor profiles. Similar to the parental tumor, the FACS profile of Wnt1-Late^Ex^ Cd49f^pos^/Epcam^neg^-injected cells contained two populations (Fig. 6B,C). A similar observation was observed for Wnt1-Late^Ex^ Cd49f^pos^/Epcam^pos^-injected cells (Fig. 6D,E). These results show that both Wnt1-Late^Ex^ populations are capable of reproducing the other population when injected into the mammary pad after FACS purification.

### Both Wnt1-Late^Ex^ tumor FACS subpopulations have activating *Hras1* mutations

To identify possible genetic drivers that might explain the difference in tumor-initiating potential, the mRNA sequence of 13 Wnt1-Early^Ex^ and 13 Wnt1-Late^Ex^ tumors was obtained to profile their mutations. It is known that a subset of MMTV-Wnt1 tumors harbor activating *Hras1* mutations (Cleary et al., 2014). Interestingly, 10 of the 13 Wnt1-Late^Ex^ tumors profiled contained either codon 12 (1 tumor)- or codon 61 (9 tumors)-activating *Hras1* mutations, whereas none of the Wnt1-Early^Ex^ tumors was mutated (Fisher's exact test *P*-value=0.0004) (Table 1). This observation is consistent with previous findings (Podsypanina et al., 2004; Cleary et al., 2014). In colorectal cancer, *APC* loss-of-function mutations synergize with *KRAS*-activating mutations to activate cancer stem cells (Moon et al., 2014). If similar synergy occurs in Wnt1-Late^Ex^ tumors, these activating *Hras1* mutations might help to explain why these tumors have tumor-initiating potential. As both Wnt1-Late^Ex^ FACS populations were capable of producing tumors when injected individually, we predicted that both populations should contain *Hras1* mutations. To test this idea, DNA was extracted from the FACS populations of two Wnt1-Late^Ex^ tumors and analyzed for the presence of *Hras1* mutations. In support of this hypothesis, both Wnt1-Late^Ex^ populations contained *Hras1* mutations.

**Table 1.**
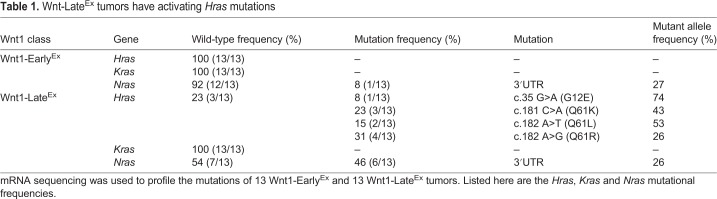
Wnt-Late^Ex^ tumors have activating *Hras* mutations

## DISCUSSION

Breast cancer is the second leading cause of cancer-related deaths in American women (American Cancer Society, 2015). Patient care is particularly complicated by breast tumor heterogeneity, which is defined by multiple intrinsic subtypes (Perou et al., 2000; Prat et al., 2010; Cancer Genome Atlas Network, 2012). Although a greater understanding of tumor biology has led to targeted treatment options for most of these subtypes (Jordan, 2003; Hynes and Lane, 2005), personalized drug targets for basal-like tumors remain an important unmet clinical need (Curigliano and Goldhirsch, 2011). Given that Wnt signaling is both enriched in basal-like tumors and predictive of poor clinical outcomes (Khramtsov et al., 2010), Wnt signaling has been proposed as an attractive drug target for these patients (Anastas and Moon, 2013). The development of effective Wnt-based therapeutics against breast tumors, however, has been slowed in part by the complexity of Wnt signaling (Anastas and Moon, 2013; Roarty and Rosen, 2010). In addition to canonical and several non-canonical pathways, Wnt signaling is also known to crosstalk with a variety of other signal transduction pathways (Collu et al., 2014; Nishita et al., 2000; Shackleford et al., 1993; Hu and Li, 2010). It is this context-dependent nature that probably accounts for the finding that paracrine Wnt signaling can induce growth of some tumors and inhibit growth in others (Green et al., 2013). Given the importance of Wnt signaling for regulating cell growth differentiation and stem cell self-renewal (Anastas and Moon, 2013), a better understanding of these signaling pathways is needed.

MMTV-Wnt1 mice are an attractive model for studying the context-dependent nature with which these aberrations influence breast tumorigenesis (Cleary et al., 2014). We recently reported that this model develops two subtypes of tumors by gene expression classification (Pfefferle et al., 2013). Given that this finding is under-represented in the literature, we sought to validate our initial observation with a more thorough examination of these two Wnt1 subtypes. Here, we show that the MMTV-Wnt1 murine model produces two phenotypically distinct subtypes of mammary tumors with unique therapeutic responses, furthering our understanding of Wnt signaling in breast cancer.

Wnt1-Early^Ex^ mice were initially characterized by their early tumor latency, accounting for ∼60% of the MMTV-Wnt1 tumors profiled in this study. In addition to having a more solid and cord-like (or trabecular) histological appearance, these tumors had areas with glandular architecture and central necrosis. These findings might help to explain why Wnt1-Early^Ex^ tumors were enriched for a hypoxia gene signature. Although these tumors contained features of both basal and luminal mammary cell types, based on immunofluorescence staining with antibodies against Krt5 and Krt8/18 markers, they also had distinct regions that did not stain positive for either cell marker. Although the results presented here are unable to address the biological impact of these non-staining regions, we propose that they are likely to be significant given that they account for ∼35% of the tumor. On a genomically defined pathway level, Wnt1-Early^Ex^ tumors exhibited higher expression of both canonical and non-canonical signaling. On a gene level, *Nkd2* and *Tgfα* were particularly highly expressed in these tumors and were capable of crosstalk with EGFR signaling (Hu and Li, 2010). We validated the therapeutic significance of this observation by treating MMTV-Wnt1 tumors with erlotinib. As predicted, Wnt1-Early^Ex^ tumors had a dynamic reduction in tumor volume after only 14 days of treatment. The FACS profile of these tumors revealed that they were ∼75% Cd49f^pos^/Epcam^neg^. Although this profile is similar to adult MaSCs (Visvader, 2009), both passaged Wnt1-Early^Ex^ tumors were unable to be serially transplanted into wild-type FVB female mice.

Wnt1-Late^Ex^ mice were initially identified by their longer tumor latency, accounting for ∼40% of the MMTV-Wnt1 tumors profiled in this study. These tumors had a bloody gross pathology, which was highlighted by the presence of ‘blood lakes’ identified by H&E staining. These tumors also had cell features of both basal and luminal mammary cell types, which account for ∼100% of the tumor. On a pathway level, Wnt1-Late^Ex^ tumors had high expression of canonical Wnt signaling compared with other mouse tumors, but lower expression in comparison with Wnt1-Early^Ex^ tumors. Although these tumors had a FACS profile that was ∼60% Cd49f^pos^/Epcam^pos^, they also contained a secondary Cd49f^pos^/Epcam^neg^ population that accounted for ∼25% of epithelial cells. Interestingly, both of these cell populations were able to give rise to new tumors when serially transplanted into wild-type FVB female mice. Similar to the parental tumor, the FACS profile of these serially transplanted cells contained two populations, indicating that both Wnt1-Late^Ex^ populations were capable of reproducing the other. This tumor-initiating property might be linked to the presence of *Hras1* mutations in Wnt1-Late^Ex^ tumors. This hypothesis was supported by the finding that *KRAS* mutations synergize with aberrant Wnt signaling in colorectal cancer to activate cancer stem cells (Moon et al., 2014). In a previous publication on MMTV-Wnt1 tumors, *Hras1* mutations were shown to be specific to the Cd49f^pos^/Epcam^neg^ population (Cleary et al., 2014), but in the tumors we sequenced *Hras1* mutations were identified in both the Cd49f^pos^/Epcam^neg^ and the Cd49f^pos^/Epcam^pos^ FACS populations. This finding was consistent with our hypothesis that these mutations help promote tumor initiation in Wnt1-Late^Ex^ tumors and might also explain why they were more resistant to EGFR inhibition.

Although the differential response rates to erlotinib in this study are intriguing and further consolidate the heterogeneity of MMTV-Wnt1-driven mammary tumors, the immediate translational significance of this observation for human triple-negative breast cancer (TNBC) patients is unknown. Specifically, EGFR inhibition has been tried in TNBC patients previously and was found to have low response rates (i.e. ∼10%); a small subset of TNBC patients did respond to the anti-EGFR antibody cetuximab (Carey et al., 2012). As a result, the availability of genomic data from responsive tumors for retrospective studies, such as comparisons to the MMTV-Wnt1 tumors published here, is limited and makes it difficult to gauge whether Wnt1-Early^Ex^ tumors are a good model for this small subset of responders without significant follow-up experiments.

The FACS profiles of Wnt1-Early^Ex^ tumors are similar to MaSCs and those of Wnt1-Late^Ex^ tumors are similar to LumProg cells. Although additional experiments (e.g. lineage tracing) will be required to unequivocally determine this, at the very least, these associations suggest which normal mammary subpopulation a given tumor most represents in its current state. If the inappropriate expansion of these cell populations was truly a stochastic event, we would not have expected to observe such a stark contrast in tumor latency between the two Wnt1 subtypes. This latency difference suggested that these are not random, but regulated events. Of the tumors profiled in this study, we did not find a single case of subtype switching (where an ‘early’ latency tumor was classified as a ‘Wnt1-Late^Ex^’ or vice versa), indicating that these regulating mechanisms are rather strong. Broadly, we hypothesize that the mechanisms governing Wnt1-Early^Ex^ tumor development are probably related to early life history and/or puberty, which occurs around this time (Visvader and Stingl, 2014). Wnt1-Late^Ex^ tumor susceptibility is probably influenced by age-related changes that increase the risk of developing *Hras1*-activating mutations, as is the case for *KRAS* mutations in colorectal cancer (Breivik et al., 1994). In summary, these data show that a classic mouse model of breast cancer is in fact heterogeneous and represented by distinct subtypes.

## MATERIALS AND METHODS

### Mouse husbandry

All animal work was carried out in University of North Carolina Division of Laboratory and Animal Medicine (UNC DLAM) facilities in compliance with Institutional Animal Care and Use Committee (IACUC) approved protocols. FVB/n mice carrying the MMTV-Wnt1 transgene were received from Harold Varmus of Memorial Sloan-Kettering (Li et al., 2000), bred and housed until the onset of a mammary tumor. The following PCR primers were used for genotyping: 5′-GGACTTGCTTCTCTTCTCATAGCC-3′ and 5′-CCACACAGGCATAGAGTGTCTGC-3′.

### Gene expression

Microarray gene expression data from 27 murine models of mammary carcinoma and normal mammary tissue were downloaded from the following gene expression omnibus (GEO) entries: GSE3165, GSE8516, GSE9343, GSE14457, GSE15263, GSE17916, GSE27101 and GSE42640 (Pfefferle et al., 2013). An additional 35 MMTV-Wnt1 tumors were microarray-profiled, as previously described (Pfefferle et al., 2013), and were uploaded to GEO under accession number GSE118164. The 420 sample data set was normalized to correct for microarray platform bias as previously described (Pfefferle et al., 2013). Gene expression signatures were created for Wnt1-Early^Ex^ and Wnt1-Late^Ex^ tumors by performing a two-class (class X versus all others or Wnt1-Early^Ex^ versus Wnt1-Late^Ex^) significance analysis of microarrays (SAM) on the microarray data set (Tusher et al., 2001). Signatures were defined as all genes highly expressed in the class of interest with a false discovery rate (FDR) of 0%. Similarly, pathway signatures were created as previously described (Pfefferle et al., 2013) (Tables S2,S3 and S4). Expression scores for each gene and pathway signature were determined by calculating the standardized mean expression of the signature within each sample.

RNA sequence libraries were prepared from 26 MMTV-Wnt1 tumors using a TruSeq RNA kit (Illumina #RS-122-2001) before being submitted to the UNC High-Throughput Sequencing Facility (HTSF) to be run on the Illumina HiSeq 2000. Reads were mapped to the Mm9 reference sequence. *Hras*, *Kras* and *Nras* mutations were identified using the Integrative Genomics Viewer (IGV) from the Broad Institute. All sequencing data were uploaded to GEO under accession number GSE118164 and Sequence Read Archive (SRA) under accession number SRP156448.

### DNA copy number

Genomic DNA was extracted from 11 Wnt1-Early^Ex^ and 10 Wnt1-Late^Ex^ tumors using a DNeasy blood and tissue kit (Qiagen #69504), labeled with a Sure Tag DNA kit (Agilent #5190-4240) and hybridized to 244K CGH microarrays (Agilent #G4415A) as previously described (Herschkowitz et al., 2012). DNA array Comparative Genomic Hybridization (aCGH) data were uploaded to GEO under accession number GSE118164.

The 21-sample aCGH data set was extracted from the UNC Microarray Database as log_2_ Cy5/Cy3 ratios, filtering for probes with Lowess normalized intensity values greater than ten in the control channel and for probes with data on greater than 70% of the microarrays (Herschkowitz et al., 2012). The probes that passed these filters where then oriented in genomic order and a ten-probe average was calculated on consecutive groups of ten probes across each chromosome, resulting in a final data set of 23,204 features. A two-class (Wnt1-Early^Ex^ versus Wnt1-Late^Ex^) SAM analysis was performed to identify genomic regions of amplification or deletion unique to each class (FDR 0%).

### Immunofluorescence

Tissue samples were fixed in 10% neutral buffered formalin (Sigma-Aldrich #HT5012) overnight before being submitted to the UNC Lineberger Animal Histopathology core facility to be paraffin embedded and sectioned. Slides were heated for 30 min at 55°C and then washed with xylene (Fisher Scientific #X3P) and ethanol (Decon Laboratories, Inc. #2716) to deparaffin the samples. To increase antigen exposure, slides were boiled for 15 min in Antigen Retrieval Citra Plus Solution (BioGenex #HK080-9K). Samples were blocked for 1 h at room temperature in TBS (BioRad #170-6435)/0.05% Tween 20 (BioRad #161-0781) plus 5% normal goat serum (Sigma-Aldrich #G9023). Proteins were labeled with murine Krt5 (Covance #PRB-160P) and murine Krt8/18 (Fitzgerald #20R-CP004) primary antibodies at 4°C overnight before being labeled with an anti-rabbit secondary antibody (Molecular Probes #A11034) and anti-guinea pig (Molecular Probes #A11076) at room temperature for 1 h. Slides were mounted with DAPI (Vector Laboratories #H-1500). Slide images were taken using a Nikon Eclipse E600 microscope and processed using ImageJ software.

### Drug treatment

From gene expression data, the Wnt1-Early^Ex^ tumor with the longest latency was 13.5 weeks and the Wnt1-Late^Ex^ tumor with the shortest latency was 16 weeks. From these observations, a 14-15 week cut-off was used to define Wnt1-Early and Wnt1-Late tumors for drug treatment analysis, as the gene expression subtype for each spontaneous tumor could not be easily determined before or after treatment. Using latency to subtype tumors, MMTV-Wnt1 tumors were randomized into treatment groups and tumor growth was monitored using two-dimensional caliper measurements (volume=[(width)^2^×length]/2) (Usary et al., 2013). Drug compounds were obtained from commercial sources (erlotinib from Genentech, Inc.) before being synthesized into chow by OpenSource Diets to a final concentration of 25 mg/kg (Usary et al., 2013). Biological inhibitors were dosed continuously for 2 weeks after primary tumors reached a width of ∼5-10 mm. The percentage change in tumor volume at the end of the 14 day treatment period was used to quantify response.

### Flow cytometry and limiting-dilution transplantation assay

MMTV-Wnt1 tumors were dissociated into a single-cell suspension using the following steps. First, each tumor was manually cut into small pieces with a razor blade in 1× collagenase/hyaluronidase (StemCell #07919) EpiCult media (StemCell #05601) before being placed in a rotator for 2 h at 37°C. Following lysis of red blood cells using ammonia chloride (StemCell #07850), the tumors were incubated in 1× trypsin-EDTA (Sigma #T4049) for 5 min at 37°C and then in a 1× Dispase (StemCell #07923) DNase I solution (StemCell #07900) for 5 min at 37°C to reduce cell clumping. Cells were filtered through a 40 µm nylon cell strainer (Fisher Scientific #08-771-1) in Hank's balanced salt solution (HBSS) media (StemCell #37150) with 10% FBS (Sigma #F2442) to obtain the final single-cell suspension. To remove non-epithelial cells, the single-cell suspension was taken through a mouse epithelial cell enrichment kit (StemCell #19758) following the manufacturer's protocol. Cells were labeled with two antibodies for 30 min at 4°C: FITC anti-mouse Epcam (eBioscience #11-5791-82) and APC anti-mouse Cd49f (eBioscience #17-0495-82). FACS was performed using a Beckman-Coulter CyAn ADP instrument and analyzed using the FlowJo v10 software program.

For limiting-dilution transplantation assays, tumors were sorted into two populations using FACS (Cd49f^pos^/Epcam^neg^ and Cd49f^pos^/Epcam^pos^), as described above, and collected into HBSS media (StemCell #37150) with 10% FBS (Sigma #F2442). Collected cells were counted using a Beckman-Coulter Z Series Coulter Counter. The appropriate number of cells were washed and resuspended in 50 µl HBSS media (StemCell #37150) with 10% FBS (Sigma #F2442) before being diluted to a final volume of 100 µl in Matrigel Matrix (Corning #354234). These cells were then injected between the right abdominal and inguinal mammary glands of five 6- to 8-week-old wild-type FVB female mice for each cell concentration. Transplanted cells were monitored for tumor growth over a 120 day period. In total, three primary MMTV-Wnt1 tumors were tested in limiting-dilution transplantation assays: two Wnt1-Early^Ex^ (182351, 6 week latency; 183984, 8 week latency) and one Wnt1-Late^Ex^ (172215, 45 week latency).

### Sanger sequencing

Genomic DNA was extracted from the FACS-purified fractions using a DNeasy blood and tissue kit (Qiagen #69504) and a portion of *Hras1* was PCR-amplified using a Taq PCR kit (Qiagen Cat #201223) with two primers: 5′-ATGGGGTATGATCCATCAGG-3′ and 5′-CACACGGAACCTTCCTCAC-3′ (Sigma-Aldrich). PCR products were enriched with a PCR purification kit (Qiagen #28104) before being submitted to the UNC Genome Analysis Facility for Sanger sequencing. Results were analyzed using Sequencher software.

## Supplementary Material

Supplementary information

## References

[DMM037192C1] American Cancer Society (2015). *Cancer Facts and Figures 2015*. Atlanta: American Cancer Society.

[DMM037192C2] Anastas J. N. and Moon R. T. (2013). WNT signalling pathways as therapeutic targets in cancer. *Nat. Rev. Cancer* 13, 11-26. 10.1038/nrc3419 23258168

[DMM037192C3] Arendt L. M., Rudnick J. A., Keller P. J. and Kuperwasser C. (2010). Stroma in breast development and disease. *Semin. Cell Dev. Biol.* 21, 11-18. 10.1016/j.semcdb.2009.10.003 19857593 PMC2823823

[DMM037192C4] Breivik J., Meling G. I., Spurkland A., Rognum T. O. and Gaudernack G. (1994). K-ras mutation in colorectal cancer: relations to patient age, sex and tumour location. *Br. J. Cancer* 69, 367-371. 10.1038/bjc.1994.67 8297737 PMC1968690

[DMM037192C5] Cancer Genome Atlas Network. (2012). Comprehensive molecular portraits of human breast tumours. *Nature* 490, 61-70. 10.1038/nature11412 23000897 PMC3465532

[DMM037192C6] Cardiff R. D., Anver M. R., Gusterson B. A., Hennighausen L., Jensen R. A., Merino M. J., Rehm S., Russo J., Tavassoli F. A., Wakefield L. M. et al. (2000). The mammary pathology of genetically engineered mice: the consensus report and recommendations from the Annapolis meeting. *Oncogene* 19, 968-988. 10.1038/sj.onc.1203277 10713680

[DMM037192C7] Carey L. A., Rugo H. S., Marcom P. K., Mayer E. L., Esteva F. J., Ma C. X., Liu M. C., Storniolo A. M., Rimawi M. F., Forero-Torres A. et al. (2012). TBCRC 001: randomized phase II study of cetuximab in combination with carboplatin in stage IV triple-negative breast cancer. *J. Clin. Oncol.* 30, 2615-2623. 10.1200/JCO.2010.34.5579 22665533 PMC3413275

[DMM037192C8] Cleary A. S., Leonard T. L., Gestl S. A. and Gunther E. J. (2014). Tumour cell heterogeneity maintained by cooperating subclones in Wnt-driven mammary cancers. *Nature* 508, 113-117. 10.1038/nature13187 24695311 PMC4050741

[DMM037192C9] Collu G. M., Hidalgo-Sastre A. and Brennan K. (2014). Wnt-Notch signalling crosstalk in development and disease. *Cell. Mol. Life Sci.* 71, 3553-3567. 10.1007/s00018-014-1644-x 24942883 PMC11113451

[DMM037192C10] Curigliano G. and Goldhirsch A. (2011). The triple-negative subtype: new ideas for the poorest prognosis breast cancer. *J. Natl. Cancer Inst. Monogr.* 2011, 108-110. 10.1093/jncimonographs/lgr038 22043054

[DMM037192C11] Fan C., Prat A., Parker J. S., Liu Y., Carey L. A., Troester M. A. and Perou C. M. (2011). Building prognostic models for breast cancer patients using clinical variables and hundreds of gene expression signatures. *BMC Med. Genomics* 4, 3 10.1186/1755-8794-4-3 21214954 PMC3025826

[DMM037192C12] Gjorevski N. and Nelson C. M. (2011). Integrated morphodynamic signalling of the mammary gland. *Nat. Rev. Mol. Cell Biol.* 12, 581-593. 10.1038/nrm3168 21829222

[DMM037192C13] Green J. L., La J., Yum K. W., Desai P., Rodewald L.-W., Zhang X., Leblanc M., Nusse R., Lewis M. T. and Wahl G. M. (2013). Paracrine Wnt signaling both promotes and inhibits human breast tumor growth. *Proc. Natl. Acad. Sci. USA* 110, 6991-6996. 10.1073/pnas.1303671110 23559372 PMC3637696

[DMM037192C14] He T. C., Sparks A. B., Rago C., Hermeking H., Zawel L., da Costa L. T., Morin P. J., Vogelstein B. and Kinzler K. W. (1998). Identification of c-MYC as a target of the APC pathway. *Science* 281, 1509-1512. 10.1126/science.281.5382.1509 9727977

[DMM037192C15] Herschkowitz J. I., Zhao W., Zhang M., Usary J., Murrow G., Edwards D., Knezevic J., Greene S. B., Darr D., Troester M. A. et al. (2012). Comparative oncogenomics identifies breast tumors enriched in functional tumor-initiating cells. *Proc. Natl. Acad. Sci. USA* 109, 2778-2783. 10.1073/pnas.1018862108 21633010 PMC3286979

[DMM037192C16] Hu T. and Li C. (2010). Convergence between Wnt-beta-catenin and EGFR signaling in cancer. *Mol. Cancer* 9, 236 10.1186/1476-4598-9-236 20828404 PMC2944186

[DMM037192C17] Hu Z., Fan C., Livasy C., He X., Oh D. S., Ewend M. G., Carey L. A., Subramanian S., West R., Ikpatt F. et al. (2009). A compact VEGF signature associated with distant metastases and poor outcomes. *BMC Med.* 7, 9 10.1186/1741-7015-7-9 19291283 PMC2671523

[DMM037192C18] Hynes N. E. and Lane H. A. (2005). ERBB receptors and cancer: the complexity of targeted inhibitors. *Nat. Rev. Cancer* 5, 341-354. 10.1038/nrc1609 15864276

[DMM037192C19] Jordan V. C. (2003). Tamoxifen: a most unlikely pioneering medicine. *Nat. Rev. Drug Discov.* 2, 205-213. 10.1038/nrd1031 12612646

[DMM037192C20] Kendrick H., Regan J. L., Magnay F.-A., Grigoriadis A., Mitsopoulos C., Zvelebil M. and Smalley M. J. (2008). Transcriptome analysis of mammary epithelial subpopulations identifies novel determinants of lineage commitment and cell fate. *BMC Genomics* 9, 591 10.1186/1471-2164-9-591 19063729 PMC2629782

[DMM037192C21] Khramtsov A. I., Khramtsova G. F., Tretiakova M., Huo D., Olopade O. I. and Goss K. H. (2010). Wnt/beta-catenin pathway activation is enriched in basal-like breast cancers and predicts poor outcome. *Am. J. Pathol.* 176, 2911-2920. 10.2353/ajpath.2010.091125 20395444 PMC2877852

[DMM037192C22] Li Y., Hively W. P. and Varmus H. E. (2000). Use of MMTV-Wnt-1 transgenic mice for studying the genetic basis of breast cancer. *Oncogene* 19, 1002-1009. 10.1038/sj.onc.1203273 10713683

[DMM037192C23] Lim E., Wu D., Pal B., Bouras T., Asselin-Labat M.-L., Vaillant F., Yagita H., Lindeman G. J., Smyth G. K. and Visvader J. E. (2010). Transcriptome analyses of mouse and human mammary cell subpopulations reveal multiple conserved genes and pathways. *Breast Cancer Res.* 12, R21 10.1186/bcr2560 20346151 PMC2879567

[DMM037192C24] Mann B., Gelos M., Siedow A., Hanski M. L., Gratchev A., Ilyas M., Bodmer W. F., Moyer M. P., Riecken E. O., Buhr H. J. et al. (1999). Target genes of beta-catenin-T cell-factor/lymphoid-enhancer-factor signaling in human colorectal carcinomas. *Proc. Natl. Acad. Sci. USA* 96, 1603-1608. 10.1073/pnas.96.4.1603 9990071 PMC15532

[DMM037192C25] Moon B. S., Jeong W. J., Park J., Kim T. I., Min do S. and Choi K. Y. (2014). Role of oncogenic K-Ras in cancer stem cell activation by aberrant Wnt/beta-catenin signaling. *J. Natl. Cancer Inst.* 106, djt373 10.1093/jnci/dju373 24491301

[DMM037192C26] Munn R. J., Webster M., Muller W. J. and Cardiff R. D. (1995). Histopathology of transgenic mouse mammary tumors (a short atlas). *Semin. Cancer Biol.* 6, 153-158. 10.1006/scbi.1995.0020 7495983

[DMM037192C27] Nishita M., Hashimoto M. K., Ogata S., Laurent M. N., Ueno N., Shibuya H. and Cho K. W. Y. (2000). Interaction between Wnt and TGF-beta signalling pathways during formation of Spemann's organizer. *Nature* 403, 781-785. 10.1038/35001602 10693808

[DMM037192C28] Nusse R. and Varmus H. E. (1982). Many tumors induced by the mouse mammary tumor virus contain a provirus integrated in the same region of the host genome. *Cell* 31, 99-109. 10.1016/0092-8674(82)90409-3 6297757

[DMM037192C29] Perou C. M., Sørlie T., Eisen M. B., van de Rijn M., Jeffrey S. S., Rees C. A., Pollack J. R., Ross D. T., Johnsen H., Akslen L. A. et al. (2000). Molecular portraits of human breast tumours. *Nature* 406, 747-752. 10.1038/35021093 10963602

[DMM037192C30] Pfefferle A. D., Herschkowitz J. I., Usary J., Harrell J. C., Spike B. T., Adams J. R., Torres-Arzayus M. I., Brown M., Egan S. E., Wahl G. M. et al. (2013). Transcriptomic classification of genetically engineered mouse models of breast cancer identifies human subtype counterparts. *Genome Biol.* 14, R125 10.1186/gb-2013-14-11-r125 24220145 PMC4053990

[DMM037192C31] Pfefferle A. D., Spike B. T., Wahl G. M. and Perou C. M. (2015). Luminal progenitor and fetal mammary stem cell expression features predict breast tumor response to neoadjuvant chemotherapy. *Breast Cancer Res. Treat.* 149, 425-437. 10.1007/s10549-014-3262-6 25575446 PMC4308649

[DMM037192C32] Podsypanina K., Li Y. and Varmus H. E. (2004). Evolution of somatic mutations in mammary tumors in transgenic mice is influenced by the inherited genotype. *BMC Med.* 2, 24 10.1186/1741-7015-2-24 15198801 PMC446228

[DMM037192C33] Prat A., Parker J. S., Karginova O., Fan C., Livasy C., Herschkowitz J. I., He X. and Perou C. M. (2010). Phenotypic and molecular characterization of the claudin-low intrinsic subtype of breast cancer. *Breast Cancer Res.* 12, R68 10.1186/bcr2635 20813035 PMC3096954

[DMM037192C34] Roarty K. and Rosen J. M. (2010). Wnt and mammary stem cells: hormones cannot fly wingless. *Curr. Opin. Pharmacol.* 10, 643-649. 10.1016/j.coph.2010.07.004 20810315 PMC2981611

[DMM037192C35] Rosen J. M. and Roarty K. (2014). Paracrine signaling in mammary gland development: what can we learn about intratumoral heterogeneity? *Breast Cancer Res.* 16, 202 10.1186/bcr3610 24476463 PMC3978850

[DMM037192C36] Santagata S., Thakkar A., Ergonul A., Wang B., Woo T., Hu R., Harrell J. C., McNamara G., Schwede M., Culhane A. C. et al. (2014). Taxonomy of breast cancer based on normal cell phenotype predicts outcome. *J. Clin. Invest.* 124, 859-870. 10.1172/JCI70941 24463450 PMC3904619

[DMM037192C37] Segditsas S. and Tomlinson I. (2006). Colorectal cancer and genetic alterations in the Wnt pathway. *Oncogene* 25, 7531-7537. 10.1038/sj.onc.1210059 17143297

[DMM037192C38] Shackleford G. M., MacArthur C. A., Kwan H. C. and Varmus H. E. (1993). Mouse mammary tumor virus infection accelerates mammary carcinogenesis in Wnt-1 transgenic mice by insertional activation of int-2/Fgf-3 and hst/Fgf-4. *Proc. Natl. Acad. Sci. USA* 90, 740-744. 10.1073/pnas.90.2.740 8380647 PMC45741

[DMM037192C39] Sharpless N. E. and Depinho R. A. (2006). The mighty mouse: genetically engineered mouse models in cancer drug development. *Nat. Rev. Drug Discov.* 5, 741-754. 10.1038/nrd2110 16915232

[DMM037192C40] Suzuki H., Toyota M., Caraway H., Gabrielson E., Ohmura T., Fujikane T., Nishikawa N., Sogabe Y., Nojima M., Sonoda T. et al. (2008). Frequent epigenetic inactivation of Wnt antagonist genes in breast cancer. *Br. J. Cancer* 98, 1147-1156. 10.1038/sj.bjc.6604259 18283316 PMC2275475

[DMM037192C41] Tusher V. G., Tibshirani R. and Chu G. (2001). Significance analysis of microarrays applied to the ionizing radiation response. *Proc. Natl. Acad. Sci. USA* 98, 5116-5121. 10.1073/pnas.091062498 11309499 PMC33173

[DMM037192C42] Usary J., Zhao W., Darr D., Roberts P. J., Liu M., Balletta L., Karginova O., Jordan J., Combest A., Bridges A. et al. (2013). Predicting drug responsiveness in human cancers using genetically engineered mice. *Clin. Cancer Res.* 19, 4889-4899. 10.1158/1078-0432.CCR-13-0522 23780888 PMC3778918

[DMM037192C43] Van Keymeulen A., Rocha A. S., Ousset M., Beck B., Bouvencourt G., Rock J., Sharma N., Dekoninck S. and Blanpain C. (2011). Distinct stem cells contribute to mammary gland development and maintenance. *Nature* 479, 189-193. 10.1038/nature10573 21983963

[DMM037192C44] Veeck J., Geisler C., Noetzel E., Alkaya S., Hartmann A., Knüchel R. and Dahl E. (2008). Epigenetic inactivation of the secreted frizzled-related protein-5 (SFRP5) gene in human breast cancer is associated with unfavorable prognosis. *Carcinogenesis* 29, 991-998. 10.1093/carcin/bgn076 18356147

[DMM037192C45] Visvader J. E. (2009). Keeping abreast of the mammary epithelial hierarchy and breast tumorigenesis. *Genes Dev.* 23, 2563-2577. 10.1101/gad.1849509 19933147 PMC2779757

[DMM037192C46] Visvader J. E. and Stingl J. (2014). Mammary stem cells and the differentiation hierarchy: current status and perspectives. *Genes Dev.* 28, 1143-1158. 10.1101/gad.242511.114 24888586 PMC4052761

[DMM037192C47] Woodward W. A., Chen M. S., Behbod F. and Rosen J. M. (2005). On mammary stem cells. *J. Cell Sci.* 118, 3585-3594. 10.1242/jcs.02532 16105882

[DMM037192C48] Zhang X., Gaspard J. P. and Chung D. C. (2001). Regulation of vascular endothelial growth factor by the Wnt and K-ras pathways in colonic neoplasia. *Cancer Res.* 61, 6050-6054. 11507052

[DMM037192C49] Zhang M., Tsimelzon A., Chang C. H., Fan C., Wolff A., Perou C. M., Hilsenbeck S. G. and Rosen J. M. (2015). Intratumoral heterogeneity in a Trp53-null mouse model of human breast cancer. *Cancer Discov.* 5, 520-533. 10.1158/2159-8290.CD-14-1101 25735774 PMC4420701

