# Supplementary material for: The MMTV-Wnt1 murine model produces two phenotypically distinct subtypes of mammary tumors with unique therapeutic responses to an EGFR inhibitor
Source: Dis Model Mech. 2019 Jul 5;12(7):dmm037192. doi: 10.1242/dmm.037192 (PMC6679375; doi:10.1242/dmm.037192)
Supplement: Supplementary information [file dmm-12-037192-s1.pdf]

Figure S1

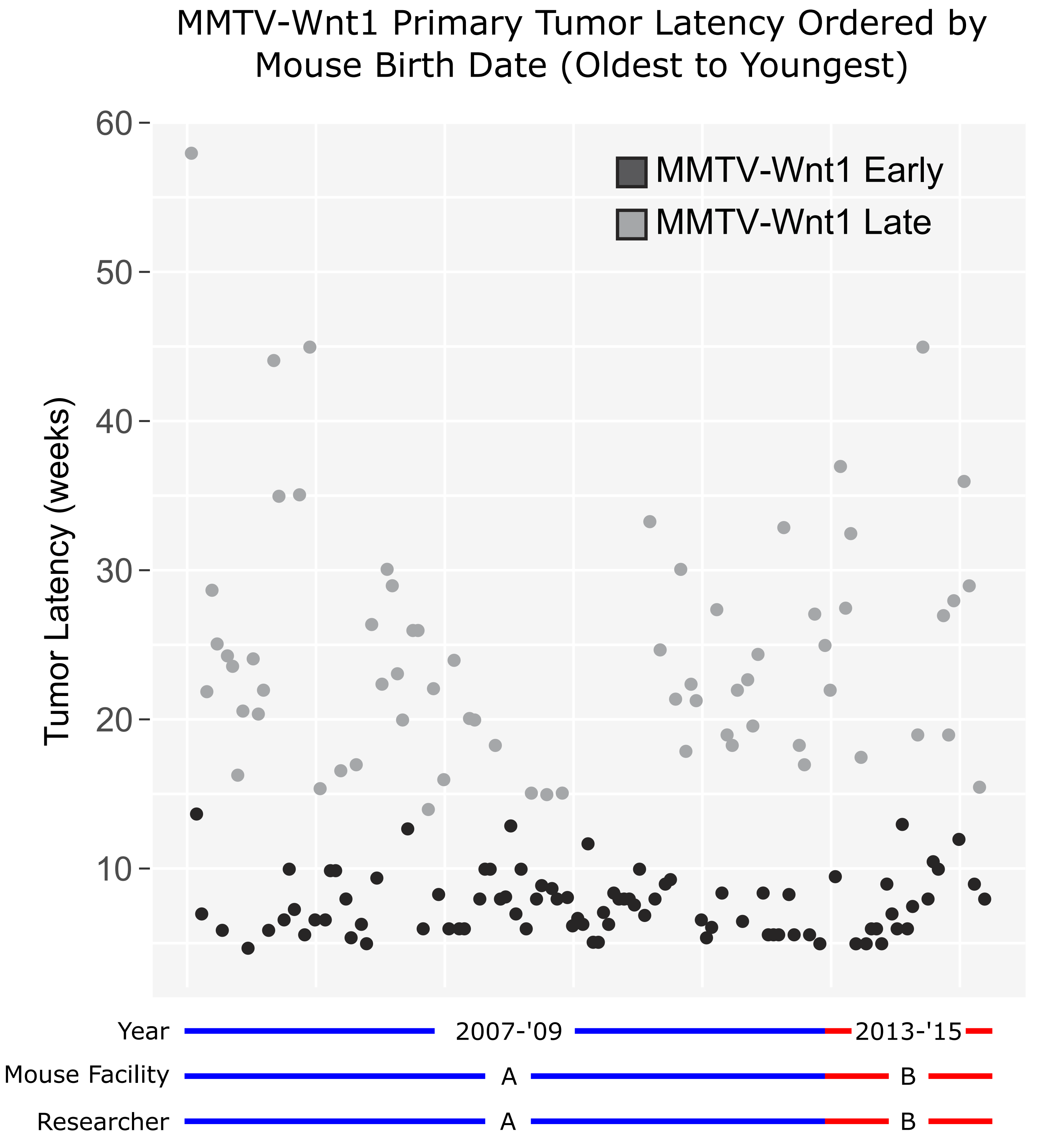

Figure S1: MMTV-Wnt1 Primary Tumor Latency Ordered by Mouse Birth Date
Displayed is a scatterplot of primary tumor latency ordered by mouse birth date with older birth dates on the left and younger birth dates on the right. Below the graph is a general breakdown of which year the mouse was born, which facility they were housed, and which researcher was the primary caregiver. Tumor latency did not correlate with birth order, mouse facility, or researcher.

Disease Models & Mechanisms • Supplementary information

Figure S2

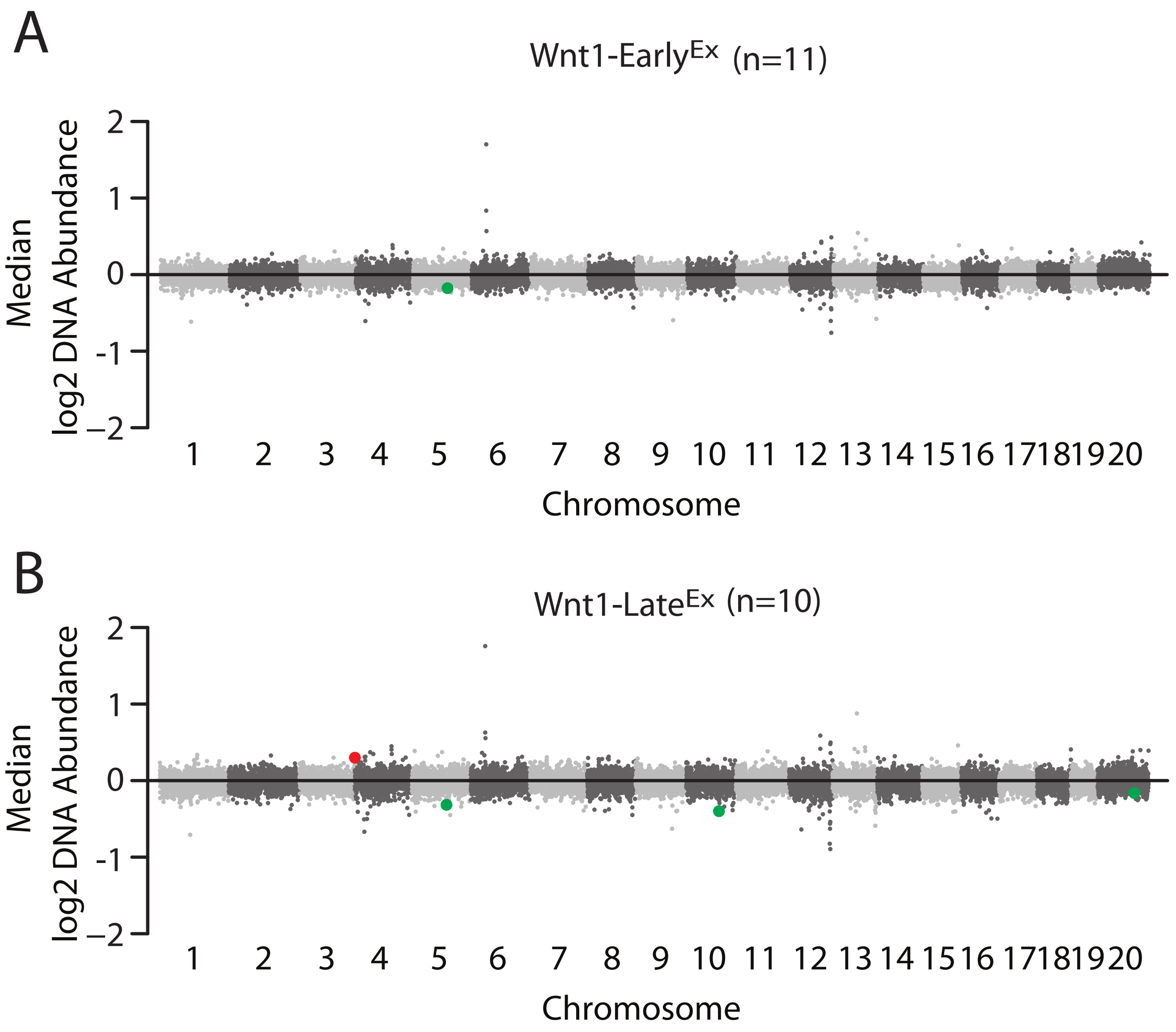

Figure S2: Wnt1-Early^{Ex} and Wnt1-Late^{Ex} have similar DNA copy number landscapes

Displayed in genomic order are the median class DNA copy number levels for **A.** 11 Wnt1-Early^{Ex} and **B.** 10 Wnt1-Late^{Ex} tumors. A two class SAM was performed between Wnt1-Early^{Ex} and Wnt1-Late^{Ex} tumors to identify class specific DNA events. Statistically significant events (FDR of 0%) are highlighted by red dots for amplified regions and green dots for deleted regions.

Figure S3

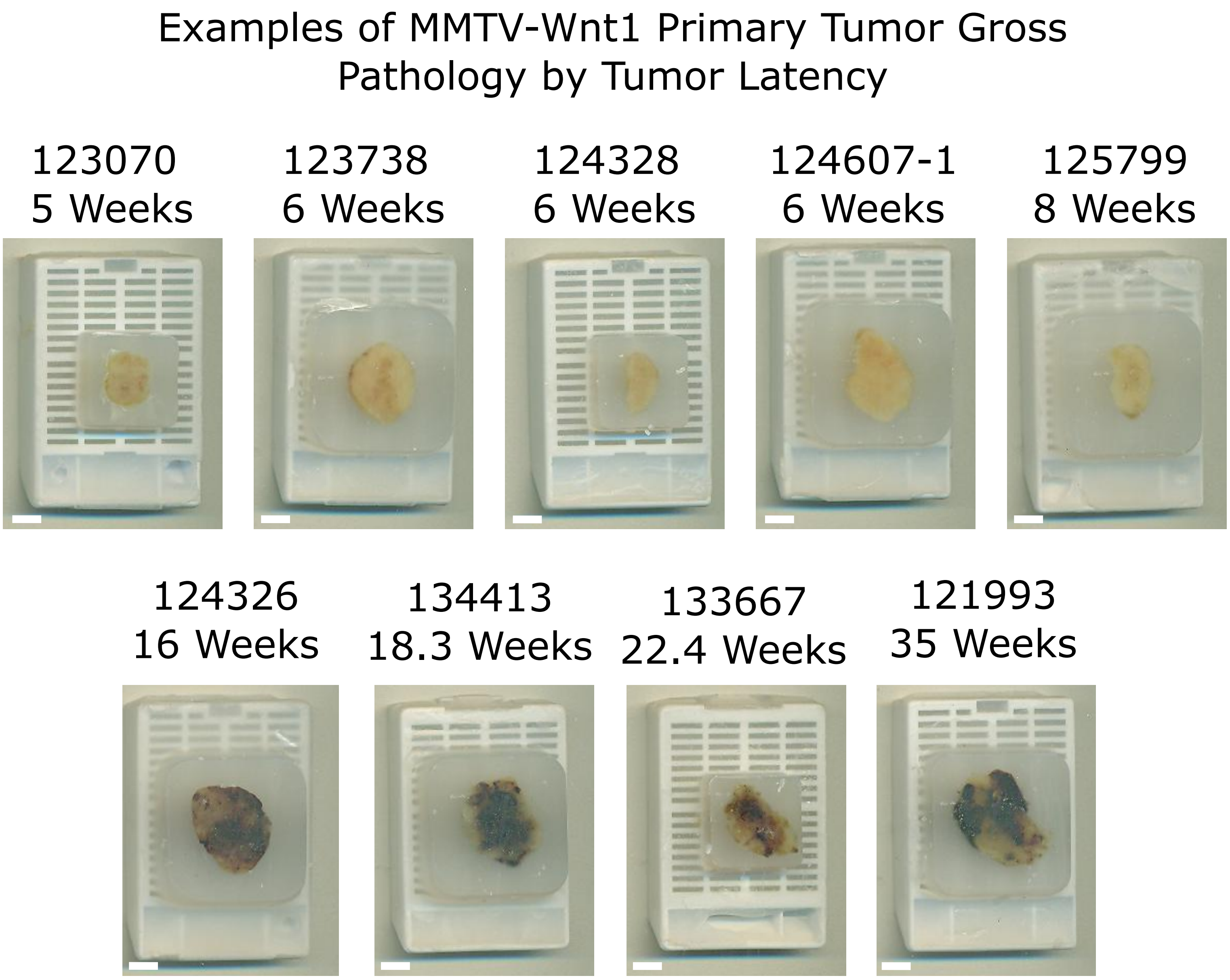

Figure S3: MMTV-Wnt1 Primary Tumor Gross Pathology by Tumor Latency
Displayed are images of nine primary MMTV-Wnt1 tumors in paraffin blocks. As visually evident, MMTV-Wnt1 tumors with later latency are more vascularized/bloodier than tumors with earlier latency. The white scale bar is 5 mm in length.

Disease Models & Mechanisms • Supplementary information

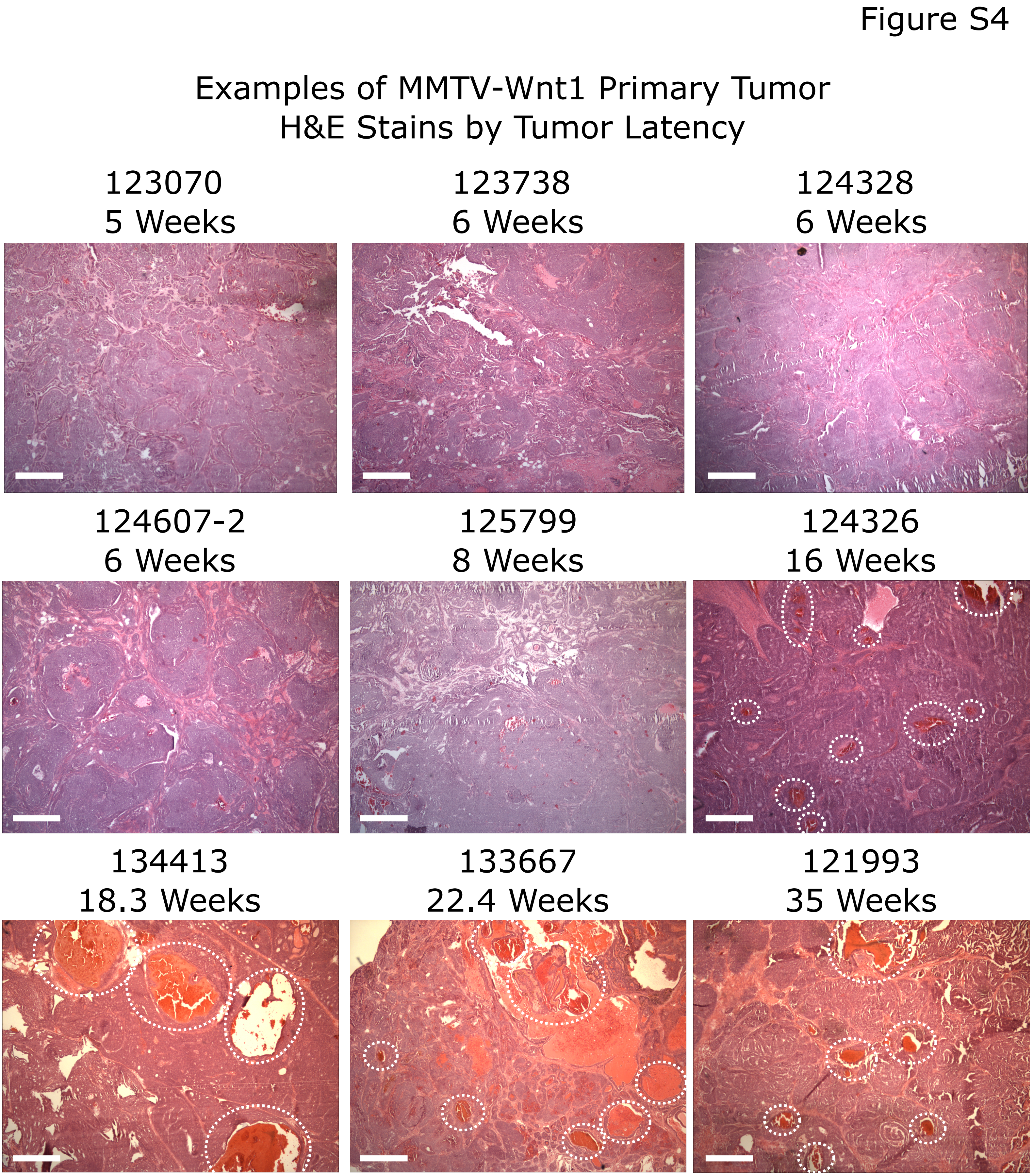

Figure S4: MMTV-Wnt1 Primary Tumor H&E Stains by Tumor Latency

Displayed are images of nine primary MMTV-Wnt1 tumors stained with H&E. As visually evident, MMTV-Wnt1 tumors with later latency have more evidence of large blood vessels (highlighted with dashed, white circles) than tumors with earlier latency. The white scale bar is 0.5 mm in length.

Disease Models & Mechanisms • Supplementary information

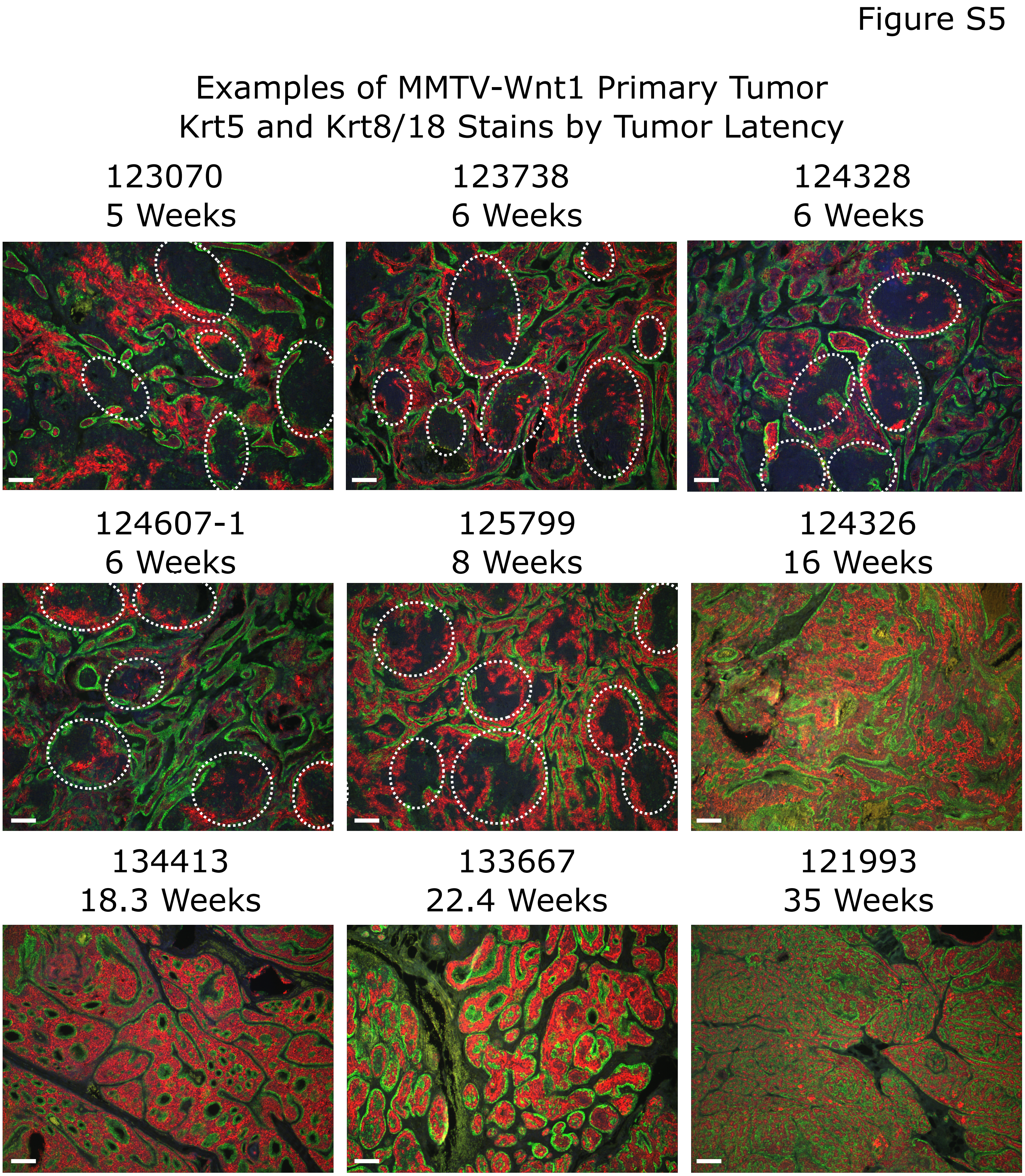

Figure S5: MMTV-Wnt1 Primary Tumor Krt5 and Krt8/18 Stains by Tumor Latency
Displayed are images of nine primary MMTV-Wnt1 tumors stained with DAPI, Krt5, and Krt8/18 antibodies. As visually evident, MMTV-Wnt1 tumors with earlier latency have more evidence of large regions that stain positive for DAPI but not Krt5 or Krt8/18 (highlighted with dashed, white circles) than tumors with later latency. The white scale bar is 0.1 mm in length.

Disease Models & Mechanisms • Supplementary information

Table S1: Pathology Review of MMTV-Wnt1 Tumor H&E Slides

Hematoxylin and eosin-stained (H&E) slides from nine MMTV-Wnt1 tumors were reviewed by a breast pathologist. The primary architectural pattern and secondary patterns or other prominent findings (e.g., blood lakes, necrosis) were recorded.

Click here to Download Table S1

Table S2: 2-Class Pathway Signature SAM (Wnt1-Early vs All Mouse Tumors)

A 2-class SAM analysis was performed comparing pathway signatures between Wnt1-Early tumors and all other mouse tumors. Pathways with higher expression in Wnt1-Early tumors have a SAM fold change greater than 1. Pathways with lower expression in Wnt1-Early tumors have a SAM fold change less than 1.

Click here to Download Table S2

Table S3: 2-Class Pathway Signature SAM (Wnt1-Late vs All Mouse Tumors)

A 2-class SAM analysis was performed comparing pathway signatures between Wnt1-Late tumors and all other mouse tumors. Pathways with higher expression in Wnt1-Late tumors have a SAM fold change greater than 1. Pathways with lower expression in Wnt1-Late tumors have a SAM fold change less than 1.

Click here to Download Table S3

Table S4: 2-Class Pathway Signature SAM (Wnt1-Early vs Wnt1-Late)

A 2-class SAM analysis was performed comparing pathway signatures between Wnt1-Early and Wnt1-Late tumors. Pathways with higher expression in Wnt1-Early tumors have a SAM fold change greater than 1. Pathways with higher expression in Wnt1-Late tumors have a SAM fold change less than 1.

Click here to Download Table S4

Table S5: Drug Treatment Data

Wnt1-Early and Wnt1-Late tumors were randomized into one of two treatment groups: untreated or erlotinib (an EGFR inhibitor). Drug treatment was initiated when primary tumors reached a width between ~5-10 mm. The percent change in tumor volume after 14 days of treatment was compared to the untreated control group to measure drug response.

Click here to Download Table S5
